# Retinal Microvascular Density and Perfusion during an Insulin-Induced Hypoglycemia Episode: A Warning Call

**DOI:** 10.1155/2023/9928582

**Published:** 2023-10-16

**Authors:** Gerardo González-Saldivar, José Gerardo González-González, David Robert Chow, Jesús Mohamed-Hamsho, Jesús Hernan González-Cortés, Adriana Sánchez-García, René Rodríguez-Gutiérrez

**Affiliations:** ^1^Department of Ophthalmology, Facultad de Medicina y Hospital Universitario “Dr. Jose E. González”, Universidad Autónoma de Nuevo León, Av. Madero y Gonzalitos s/n, Colonia Mitras Centro, Monterrey, Nuevo Leon 64460, Mexico; ^2^Plataforma INVEST Medicina UANL-KER Unit Mayo Clinic (KER Unit México), Universidad Autónoma de Nuevo León, Calle Dr. Eduardo Aguirre Pequeño s/n, Sotano Edificio CRIDS, Colonia Mitras Centro, Monterrey, Nuevo Leon 64460, Mexico; ^3^Endocrinology Division, Facultad de Medicina y Hospital Universitario “Dr. Jose E. González”, Universidad Autónoma de Nuevo León, Av. Madero y Gonzalitos s/n, Colonia Mitras Centro, Monterrey, Nuevo Leon 64460, Mexico; ^4^Department of Ophthalmology, St. Michael's Hospital, Toronto, ON, Canada; ^5^Knowledge and Evaluation Research Unit in Endocrinology, Mayo Clinic, Rochester, MN 55905, USA

## Abstract

**Aim:**

To evaluate retinal vascular perfusion and density by optical coherence tomography angiography (OCTA) before, during, and after hypoglycemia in individuals with diabetes mellitus with or without diabetic retinopathy (DR).

**Methods:**

A focused clinical history was performed, followed by an ophthalmological examination to document retinopathy status. OCTA was performed at baseline, at hypoglycemia, and at glucose normalization. Eye tracking and eye alignment devices on the platform were used to obtain a macular thickness cube (512 × 128) and vascular perfusion and density protocols of 3 × 3 mm. Retinal vascular reactivity was analyzed with superficial plexus vascular perfusion and density protocols on OCTA.

**Results:**

Fifty-two participants encompassing 97 eyes fulfilled the eligibility criteria. Their mean age was 42.9 ± 15.1 years (range, 22 to 65), and 20 (38.2%) were men. We found a statistically significant difference in vascular perfusion and density when comparing all groups at baseline. The controls had higher vascular perfusion and density values than the cases. Vascular perfusion and density were significantly reduced in all groups during the hypoglycemia episode, except for vascular density in DR cases.

**Conclusion:**

Acute hypoglycemia significantly alters the retinal vascularity in DM patients with and without DR, suggesting that repeated episodes of acute hypoglycemia could exacerbate retinopathy in the long term.

## 1. Introduction

The severity and duration of hyperglycemia are strongly associated with the risk of diabetic retinopathy (DR), nephropathy, and neuropathy [1]. The molecular changes behind this “microvascular diabetic triopathy” have been well elucidated in many *in vitro* and *in vivo* animal and human studies [2, 3]. Recognition of specific abnormal pathways, such as vascular endothelial growth factor overexpression in proliferative DR, has resulted in directed therapies that have improved the management and prognosis of this complication [4]. Interrupting these metabolic disturbances by achieving glucose normalization with tight glycemic control explains the prevention or delay of microvascular complications found in multiple clinical trials [5, 6]. However, setting tight glycemic goals has not been without harm or controversy [7]. Hypoglycemia is the most common complication of tight glycemic control associated with morbidity and mortality in the brain and heart, among others. Furthermore, recent studies have suggested hypoglycemia as a potential mechanism for developing and exacerbating diabetic macro- and microvascular complications [8–10].

Some observational and retrospective studies have identified multiple severe hypoglycemia episodes as a predictive factor for DR development and worsening [11, 12]. Known diabetic microvascular complications have been identified as aggravating factors due to hypoglycemia [10]. Studies in subjects with type 1 diabetes have documented that acute hypoglycemia decreases retinal responses to stimuli and increases retinal neurodegeneration [13, 14]. *In vivo* studies evaluating the retinal vascular repercussion during an episode of acute hypoglycemia and after glucose normalization in subjects with and without diabetes have not, to our knowledge, been carried out.

Therefore, we performed a comparative, self-controlled, and blinded study evaluating, as a primary endpoint, retinal microvascular changes (vasculature perfusion and density) by optical coherence tomography angiography (OCTA) before, during, and after controlled insulin-induced hypoglycemia in type 1 or 2 diabetes mellitus (DM) patients with or without DR and controls.

## 2. Materials and Methods

### 2.1. Participants

The Institutional Review Board and Ethics Committee approved this study, which adhered to the Declaration of Helsinki provisions. All participants provided written informed consent before enrollment. Participants were recruited from the Endocrinology Division of our university hospital. Cases were men and women aged from 18 to 65 diagnosed with type 1 or 2 diabetes. General ophthalmic examination results were reviewed in hospital records. Exclusion criteria were participants with a clinical diagnosis of ischemic heart disease, history of seizures, untreated endocrinological disorders, high blood pressure, and any fundus disease that may lead to structural, functional, or morphological changes of the macula (myopia higher than −6.00 diopters or BCVA worse than 20/40). Participants who did not reach hypoglycemia after two insulin infusions separated by 45 minutes were eliminated; OCTA scans with an image quality score <7 and motion, segmentation, or projection artifacts were also eliminated to avoid misinterpretation. Cases were also divided into two groups according to the presence or absence of DR, while controls were healthy adults.

### 2.2. Study Protocol and Measurements

On the day of the intervention, all participants attended the Endocrinology Division in the morning after a 10-hour fast. A clinical history focused on glycemic and other metabolic and clinical goals of diabetes, and other comorbidities were performed and followed by an ophthalmological examination to document retinopathy status. After clinical examination, a baseline OCTA was performed with Cirrus HD Angioplex equipment (Carl Zeiss Meditec, Dublin, CA). We first evaluated the right eye and then the left, according to our standardized ophthalmological study protocol. The time between OCTA examinations was 60–90 seconds. Eye tracking and eye alignment devices on the platform were used to obtain a macular thickness cube (512 × 128) and vascular perfusion and density protocols of 3 × 3 mm. The angiometric software of the Cirrus HD-OCT automatically calculated the inner boundary of the superficial capillary plexus as the internal limiting membrane and the outer boundary as the inner border of the inner plexiform layer. Vessel area density reflects the proportion of the OCT angiogram occupied by vessels (white pixels) and the avascular area (black pixels) and was defined as the proportion of the white pixels divided by the total pixels in the image. Vascular perfusion was measured by analyzing the changes in the scattering of light caused by moving blood cells within blood vessels [15].

After the baseline examination, an insulin tolerance test was performed on each participant to induce hypoglycemia. A 22-gaude catheter was placed in an antecubital vein to ensure adequate test handling. An intravenous bolus dose of short-acting insulin at 0.15 units/kg was initially administered. During the test, OCTA and capillary and plasma glucose samples were obtained every 10 minutes. Plasma glucose was determined with the glucose oxidase method (Pointe Scientific Inc, USA), while a portable glucometer (Accu-Chek Performa, Roche, Germany) was used for capillary glucose. Hypoglycemia was defined as <3.1 mmol/L (55 mg/dL) by capillary glucose measurement associated or not with symptoms of hypoglycemia. Five minutes after the patients presented hypoglycemia by capillary measurement, with or without clinical findings, under very strict vigilance, intravenous glucose (12.5 mL, 50% glucose solution) was administered until euglycemia was achieved. Participants remained in observation for 6 hours after the protocol. If hypoglycemia was not reached after 45 minutes, the initial dose was repeated, followed by the same protocol. If the participant did not reach hypoglycemia, there were no further interventions. OCTA was performed at baseline, at hypoglycemia (3–5 min after confirmation by glucose levels), and at glucose normalization (10 min after IV glucose administration) for further analysis. Retinal vascular reactivity was analyzed with superficial plexus vascular perfusion and density protocols on OCTA.

### 2.3. Statistical Analysis

Numerical variables are reported as means and standard deviations or median and interquartile range; categorical variables are reported as frequencies and percentages. Normality was determined using the Kolmogorov–Smirnov test. Categorical variables were compared using Pearson's *χ*^2^ test or Fisher's exact test for 2 × 2 tables. An unpaired Student's *t*-test or the Mann–Whitney *U* test was used to compare continuous variables according to normality. When more than two groups were compared, one-way ANOVA or the Kruskal–Wallis test was performed. Bonferroni correction was used as a post hoc test. The repeated measures ANOVA test evaluated in-group differences during the OCTA study. Statistical analysis was performed in SPSS Statistics version 25 (IBM Corp., Armonk, NY). A *P* value <0.05 was considered statistically significant.

## 3. Results

### 3.1. Demographic Characteristics

We evaluated 71 subjects; 19 were excluded because of one or more exclusion or elimination criteria. A total of 52 participants, encompassing 97 eyes, fulfilled all eligibility criteria for our study. The baseline demographics of our study population are summarized in Table 1. Patients had a mean age of 42.9 ± 15.1 years (range 22–65); 20 (38.2%) participants were men.

Cases with DR (11 subjects) were similar in age to cases without DR (47.1 ± 12.5 *vs.* 47 ± 14.3 yrs.), but they were older than controls (36.7 ± 15.7 yrs.). Obesity was the most frequent BMI category in both groups of cases. A self-reported hypertension diagnosis was higher in cases with DR (42.9%) than those without DR (23.1%), while dyslipidemia was similar in these groups. Smoking prevalence was higher in cases without DR and similar between healthy controls and cases with DR. Cases with DR had longer diabetes durations (13.0, 4–19 yrs.) than cases without DR (7.0, 3–12.2 yrs.). A major proportion of cases with DR used insulin (57.1%) and had severe hypoglycemia events in the year before this study (71.4%) compared to cases without DR (30.8%). We also identified 3 out of 11 cases with DR in which only one eye was affected. No adverse events related to severe hypoglycemia were observed. However, around 85% of participants with and without DM referred common hypoglycemia symptoms, such as headache, dizziness, and feeling tired and hungry.

### 3.2. OCTA Assessments between Groups

**Figure 1:**
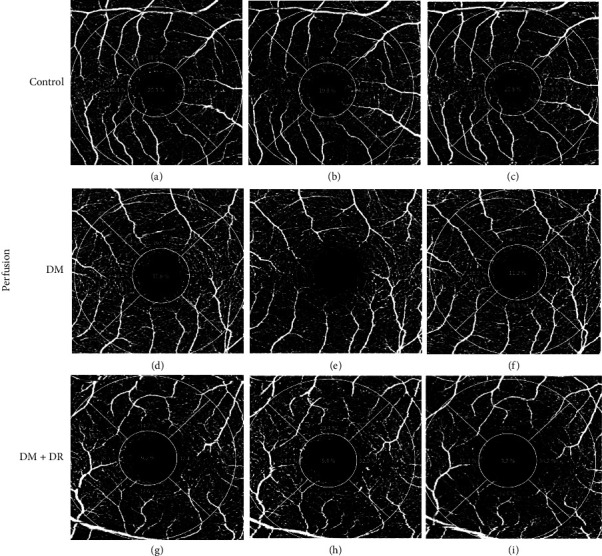
3.0 × 3.0 mm mode OCTA images of retinal perfusion changes. (a–c) SCP retinal perfusion in control patients. (d–f) SCP before, during, and after hypoglycemia in DM without DR. (g–i) SCP retinal perfusion in before, during, and after hypoglycemia in DM with DR. SCP: superficial capillary plexus; DM: diabetes mellitus; DR: diabetic retinopathy. (a) SCP: Total Perf 37.4%, (b) SCP: Total Perf 36.5%, (c) SCP: Total Perf 36.8%, (d) SCP: Total Perf 37.1%, (e) SCP: Total Perf 34.2%, (f) SCP: Total Perf 36.7%, (g) SCP: Total Perf 32.7%, (h) SCP: Total Perf 27.6%, and (i) SCP: Total Perf 28.7%.

**Figure 2:**
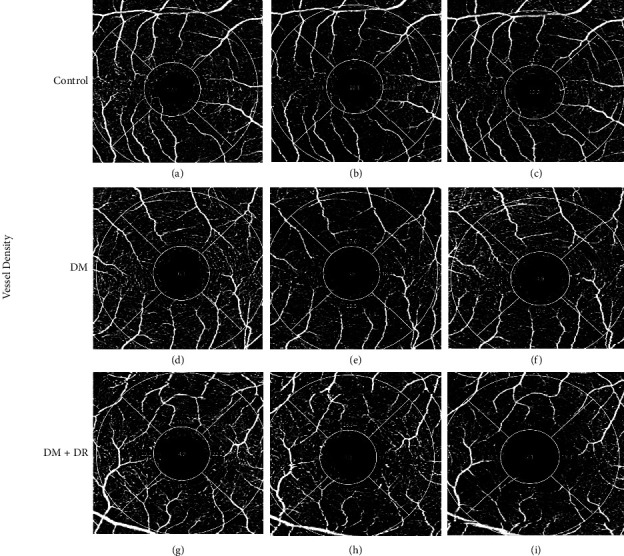
3.0 × 3.0 mm mode OCTA images of retinal vessel density. (a–c) SCP retinal perfusion in control patients. (d–f) SCP before, during, and after hypoglycemia in DM without DR. (g–i) SCP retinal perfusion in before, during, and after hypoglycemia in DM with DR. SCP: superficial capillary plexus; DM: diabetes mellitus; DR: diabetic retinopathy. (a) SCP: total VD 21.9%, (b) SCP: total VD 19.6%, (c) SCP: total VD 21.2%, (d) SCP: total VD 20.8%, (e) SCP: total VD 16.3%, (f) SCP: total VD 18.4%, (g) SCP: total VD 18.2%, (h) SCP: total VD 14.9%, and (i) SCP: total VD 15.4%.

A statistically significant difference in superficial plexus vascular perfusion values was observed (*P* *=* 0.009) comparing all groups at baseline (Table 2 and Figure 1). The post hoc analysis revealed no statistical difference between healthy participants, cases without DR, or cases with and without DR. However, a statistically significant difference between controls and cases with DR was revealed, observing lower perfusion values in the latter (34.58 ± 3.5 vs. 31.08 ± 4.38, respectively; *P* *=* 0.01). A similar statistically significant difference was observed for vascular density values among the three groups at baseline (controls 19.62 ± 1.46; DM without DR 18.54 ± 2.02; DM with DR 16.85 ± 3.28; *P* *=* 0.002). The post hoc analysis only revealed a statistically significant difference between the superficial plexus vascular density values of controls and cases with DR at baseline (19.62 ± 1.46 vs. 16.85 ± 3.28, respectively; *P* *=* 0.002). Controls had higher superficial plexus vascular perfusion and density values than cases. Cases with DR had the lowest superficial plexus vascular perfusion and density values than the controls and cases without DR (Table 2 and Figures 1 and 2).

### 3.3. OCTA Assessment during the Hypoglycemic Event

Figures 1 and 2 show the retinal perfusion and vessel density changes before, during, and after hypoglycemia in healthy controls and DM patients with and without DR. Superficial plexus vascular perfusion and density were individually evaluated among groups during the hypoglycemia episode (Table 2). A statistically significant reduction of vascular perfusion values was observed for each group during hypoglycemia: healthy controls (34.58 ± 3.5 to 32.42 ± 3.74; *P* *=* 0.008), cases without DR values (33.54 ± 2.79 to 31.71 ± 3.77; *P* *=* 0.007), and cases with DR values (31.08 ± 4.38 to 29.46 ± 4.46; *P* *=* 0.001). Of all the groups, DR cases had the lowest vascular perfusion values and the greatest decrease compared to baseline. On average, vascular perfusion was higher in controls than in DM cases after the hypoglycemic episode. As the hypoglycemia resolved and euglycemia was reached, vascular perfusion returned to near-normal levels in all groups; still, the group of cases with DR had the lowest perfusion values and the highest difference from baseline perfusion values (Table 2).

When the vascular density response to hypoglycemia was evaluated, a statistically significant reduction was observed in healthy controls (19.62 ± 1.46 to 18.46 ± 2.32; *P* *=* 0.041) and DM cases without DR (18.54 ± 2.02 to 17.73 ± 2.58; *P* *=* 0.016). As euglycemia was achieved, such as vascular perfusion, the same tendency to return to baseline values was observed; recurrently, cases with DR had the lowest density values at baseline (Table 2). Also, cases with DR showed the lowest recovery capacity when returning to normal glycemic values.

## 4. Discussion

### 4.1. Main Findings

This self-controlled, blinded, comparative study found that acute hypoglycemia events reduce the retina's vascular density and perfusion in patients with diabetes with or without DR and healthy controls. As could be expected, patients with DR at hypoglycemia presented significantly lower vascular perfusion and density values than patients without DR and healthy controls. As previously reported, patients with DR had more years of disease evolution [16] and a greater prevalence of severe hypoglycemic events in the last year. Many were under insulin treatment compared to patients without DR.

### 4.2. Comparison with Previous Studies

Zhao et al. conducted a meta-analysis of cohort studies of patients with type 2 diabetes. They reported that initiating insulin was associated with an increased risk for DR [17]. They attributed this finding to probable hypoglycemia events, secondary to initiating an intensive glycemic control, suggesting exploring this possibility further. More recently, Yang et al. examined the retinal microvascular changes and associated factors in T2DM patients before and after intensive insulin therapy [18]. Similar to our findings, they determined a decrease in vessel density in the superficial capillary plexus after intensive insulin treatment, supporting adverse effects on the retinal microcirculation.

Current guidelines do not disclose any algorithm, protocol, or specific recommendation to minimize the frequency of hypoglycemia in patients treated with insulin or antidiabetic agents [19]. Moreover, mild hypoglycemia episodes are often unrecognized by patients or relatives nor reported to physicians, leaving the true prevalence underreported [20]. Epidemiological studies have associated severe hypoglycemia episodes as an independent risk factor for DR development and progression in type 1 and 2 diabetes [11, 12]. Tanaka et al. studied a cohort of 1,221 T2DM patients for 8 years. After adjusting for confounding factors, the severe hypoglycemia HR was 4.35 (95% CI 1.98–9.56; *P* < 0.01) for DR incidence [12]. Diallo et al. studied a cohort of 712 T1DM patients for one year and reported from a multivariate regression that severe hypoglycemia HR for any DR was 3 (CI 1.99–4.52; *P* < 0.001) and for proliferative DR was 3.67 (CI 2.74–5.25; *P* < 0.001) [11]. Recurrent acute hypoglycemia has been proposed as a factor that harms the retina directly. Di Leo et al. demonstrated a nonselective loss of contrast sensitivity in type 1 diabetes patients with tight glycemic control who had reported recurrent hypoglycemia episodes [21]. They concluded that repeated hypoglycemia episodes could be responsible for the observed preretinopathy deficit in these patients. Furthermore, hypoglycemia has been suggested to affect the microvasculature, aggravating small blood vessels with established defects previously harmed by hyperglycemia-related mechanisms [9]. Recently, this finding was proven in several animal models [22–25]. Hypoglycemia is associated with lower vitreous glucose concentrations and lower survivability of the retina under ischemic conditions [24]. Furthermore, Yoshinaga et al. described in a DM-mice model that hypoglycemia is associated with an increased VEGF expression in the retina [26]. These animal models could explain the mechanism behind the acute changes in the retina's vascularity associated with hypoglycemia events observed in our study. Several theories, such as the osmotic force theory, synergistic hypothesis, blood-retinal barrier breakdown, and VEGF hypothesis, have been proposed to explain diabetic retinopathy after a large and fast reduction in glucose levels achieved in tight glucose control [27].

The prevalence of insulin-induced hypoglycemia in type 1 or 2 DM depends on many factors, such as diabetes education, dietary adherence, exercise patterns, type of antidiabetic medications, diabetic chronic microvascular complications, drug interactions, and goals of diabetes management. Their interaction determines the characteristics of the hypoglycemia, such as mild or severe, fast or slow, prolonged or brief, and recognized or unrecognized [28]. There is no question that in all diabetic patients, many episodes are not clearly identified or even unrecognized over time [29]. As a consequence, the prevalence of hypoglycemia in type 1 or type 2 diabetes is variable and frequently underreported. The introduction of continuous interstitial glucose measurements has shown that hypoglycemic events are much more common than anticipated. The use of these tools to assess the quality of glycemic control and detect the presence of mild to severe unrecognized hypoglycemia could alert the physician to be cautious of the potential for DR progression. It is worth mentioning that controlled hypoglycemic events in our study were mild and brief and decreased retinal vascular density and perfusion. It is acceptable to speculate that severe and prolonged hypoglycemic episodes could have a more deleterious effect on the retina without reversibility or progression of our findings on the retina. In normal conditions, autoregulation maintains ocular blood homeostasis and allows adjustments according to metabolic changes, such as hypoglycemia. Nevertheless, impaired autoregulation of ocular blood flow has been described in the pathophysiology of eye vascular diseases, such as open-angle glaucoma and DR [30]. Although autoregulation may be involved in the recovery of perfusion of the retina in our DR cases, it is likely to consider a loss of the retinal capacity to adjust in response to systemic metabolic factors.

Although clinical evidence of retinal damage or progression of DR is still hypothetical, our findings and other studies suggest caution with hypoglycemia, and specific recommendations to avoid it should be included in diabetes management goals. Assessment of the retinal vascular perfusion and density by OCTA in subjects in a prolonged coma due to severe hypoglycemia is lacking and is an attractive issue. This clinical scenario may magnify the consequences of hypoglycemia on DR and help us to understand its consequences on retinal function and morphology.

### 4.3. Implications for Clinical Practice and Research

While our study does not demonstrate a progression of DR due to the nature of its design, we addressed one of the main pathophysiological mechanisms behind DR progression (reduced retinal vascular density and perfusion) that leads to blindness and a decrease in visual acuity. The results of our study build up evidence regarding the need to avoid hypoglycemia and be cautious regarding the use of insulin and other antidiabetic agents with hypoglycemic potential (e.g., sulfonylureas), particularly in patients with diabetes seeking tight glycemic control. In addition, clinical trials or at least cohort studies comparing retinal findings in patients with and without recurrent hypoglycemia events may also clarify the clinical implications of this association in participants with or without DR.

### 4.4. Strengths and Limitations

Our study has some limitations. First, due to its methodological design, it cannot show the impact that acute changes in the retina vascularity secondary to induced hypoglycemia could have in the development and progression of DR. Moreover, it does not assess the impact of more frequent, profound, and prolonged hypoglycemia on the retina. Nevertheless, this is the first to uncover a vascular impairment in human retina vessels secondary to acute hypoglycemia episodes, with rapid recovery after blood glucose normalization. All participants were subjected to a controlled setting of hypoglycemia. Second, we did not categorize DR patients by stage. However, our study was designed as a proof of concept to compare healthy controls and DM cases. Current results are valuable to further explore changes in vascular density and perfusion in different DR stages, including an appropriate sample size for each subgroup. Although our outcomes are limited to those found in the superficial retina plexus, the deep retina plexus and the external retina avascular zone, irrigated by the outer choroid, have repeatedly been shown to be more prone and susceptible to ischemic fluctuations. Our findings might extrapolate or even be worse in the outer retina plexus although vasculature changes due to hypoglycemia should be assessed in all retina plexus. Further studies could elucidate the effect that recurrent (recognized and unrecognized), mild to moderate, and prolonged hypoglycemia episodes have on the long-term development and progression of DR. Once this association is strongly proven, its prevention could be easily answered by continuous blood glucose sensors that recognize and alert when the patient is near to low blood glucose levels.

## 5. Conclusions

For over a half-century, the clinical focus on diabetes management has been tight glucose control minimizing hyperglycemia to avoid or retard DR and other microvascular and macrovascular complications. On the other hand, hypoglycemia in tight glucose control has been considered a light statement to avoid neurological or cardiovascular implications but not in relation to the development or progression of chronic microvascular complications, such as DR. Our study showed impairment in vascular density and perfusion during acute induced hypoglycemia. These findings deserve attention, and this link should be assessed in real life in DM patients. These individuals have many undetected and less frequently detected hypoglycemia episodes that may increase the risk of DR progression in the long term. Further studies should elucidate this possibility. If demonstrated, this could put hypoglycemia avoidance and tight glycemic control as our most cost-effective tools to avoid DR development and progression.

## Figures and Tables

**Table 1:** Demographic and clinical characteristics.

	Healthy participants (*n* = 21)	DM without DR (*n* = 20)	DM with DR (*n* = 11)
Age, years (mean, SD)	36.79 (15.76)	47 (14.3)	47.71 (12.51)
Female gender (*n*, %)	12 (57.1)	12 (61.5)	8 (71.4)
BMI (mean, SD)	28.63 (4.02)	30.27 (5.9)	28.18 (3.42)
BMI, groups (*n*, %)			
Normal	5 (21.4)	3 (15.4)	3 (28.6)
Overweight	8 (42.9)	5 (23.1)	3 (28.6)
Obesity	7 (35.7)	12 (61.5)	5 (42.9)
Hypertension diagnosis (*n*, %)	3 (14.3)	5 (23.1)	5 (42.9)
Dyslipidemia diagnosis (*n*, %)	0 (0)	9 (46.2)	5 (42.9)
Regular cigarette smoker (*n*, %)	3 (14.3)	5 (23.1)	2 (14.3)
Diabetes-related variables			
Years of diagnosis (median, IQR)	DNA	7 (3–12.25)	13 (4–19)
Diabetic nephropathy (*n*, %)	DNA	0 (0)	0 (0)
Diabetic neuropathy (*n*, %)	DNA	8 (38.5)	5 (42.9)
Major adverse cardiovascular events (*n*, %)	DNA	0 (0)	0 (0)
Insulin use (*n*, %)	DNA	6 (30.8)	6 (57.1)

SD: standard deviation; BMI: body mass index; IQR: interquartile range; DM: diabetes mellitus; DR: diabetic retinopathy; NA: not applicable.

**Table 2:** Vascular reactivity to hypoglycemia event.

3 × 3 mm protocol	Vascular perfusion (%)				Vascular density (%)			
Stages	Healthy (*n* = 42)	DM without DR (*n* = 37)	DM with DR (*n* = 18)	*P*value	Healthy (*n* = 42)	DM without DR (*n* = 37)	DM with DR (*n* = 18)	*P*value
Baseline	34.58 (3.5)	33.54 (2.79)	31.08 (4.38)	**0.009**	19.62 (1.46)	18.54 (2.02)	16.85 (3.28)	**0.002**
Hypoglycemia	32.42 (3.74)	31.71 (3.77)	29.46 (4.46)	0.095	18.46 (2.32)	17.73 (2.58)	16.08 (3.04)	**0.034**
Return to normoglycemia	33.83 (2.74)	33.08 (4.1)	30.15 (4.24)	**0.016**	19.17 (1.6)	18.42 (2.5)	16.38 (2.98)	**0.004**
*P* value	**0.011**	**0.011**	**0.001**		**0.041**	**0.016**	*0.094*	

SD: standard deviation; IQR: interquartile range; DM: diabetes mellitus. Bold values represent the statistically significant values (*P* < 0.05).

## Data Availability

The data used to support the study are available from the corresponding author upon reasonable request.
